# Analysis of the Vehicle Chassis Axle Fractures

**DOI:** 10.3390/ma16020806

**Published:** 2023-01-13

**Authors:** Živilė Decker, Vitalijus Rudzinskas, Kazimierz Drozd, Jacek Caban, Jurijus Tretjakovas, Aleksander Nieoczym, Jonas Matijošius

**Affiliations:** 1 Department of Mechanical and Material Engineering, Faculty of Mechanics, Vilnius Gediminas Technical University, J. Basanavičiaus g. 28, LT-03224 Vilnius, Lithuania; 2 Faculty of Mechanical Engineering, Lublin University of Technology, 20-618 Lublin, Poland; 3 Department of Applied Mechanics, Faculty of Civil Engineering, Vilnius Gediminas Technical University, Saulėtekio al. 11, LT-10223 Vilnius, Lithuania; 4 Mechanical Science Institute, Vilnius Gediminas Technical University, J. Basanavičiaus g. 28, LT-10223 Vilnius, Lithuania

**Keywords:** failure analysis, FEA, fracture mechanics, macroscopic research, semi-trailers

## Abstract

With the rapid development of the road transport industry, trucks with semi-trailers have become the main means of transporting goods by road. High quality, durability and reliability of the construction are the main requirements for the production of trailers. Trailer and semi-trailer axles are one of the main and most important components of a truck. Due to the fact that semi-trailer axles are subjected to additional static and dynamic loads during operation, their proper construction is extremely important, therefore they should be carefully designed and tested. The durability of the suspension components refers to the duration of the onset of fatigue. This article presents an analysis of damage to the rear axle of the semi-trailer using macroscopic observations of the damage site and dynamic FEA of stress distribution in the axle material. In order to identify the probable cause of the damage, eight cases of loading the semi-trailer axle were considered. Analytical solutions have shown that in various cases the yield point is exceeded and the strength limit of the modeled semi-trailer axle is reached. The risk of damage to the vehicle’s suspension system components increases on poor roads (bumps and winding road sections).

## 1. Introduction

With the rapid development of the road transport industry, trucks with semi-trailers have become the main means of transporting goods by road. The economic goal of all transport companies is to transport as many goods as possible and with as few journeys as possible. High quality, durability, and structural reliability are the main requirements for the production of trailers. The axles of trailers and semi-trailers are one of the main and most important elements, which must be carefully designed and tested experimentally under static and dynamic loads, as the axle is subjected to additional loads in the event of road roughness or off-road [1]. The durability of the suspension parts refers to the duration of the onset of fatigue, defined as the number of cycles up to a certain component cracking length under cyclic loads [2,3].

The reliability of individual safety systems of a given means of transport translates directly into road traffic safety [4,5,6,7,8]. Much attention is also paid to the diagnostics of individual vehicle systems that affect safety. It is worth mentioning here the research presented by Savchenko et al. [9] and Gnap et al. [10], who demonstrated the possibility of using MEMS sensors in vehicles. Hudec et al. [11], Gajek [12] and Tucki et al. [13] paid attention to the monitoring of the technical condition of vehicles. On the other hand, research on the impact of heavy goods vehicle load during braking is presented in detail in [14,15,16,17].

The topic of scientific information is the determination of the causes of vehicle chassis axle defects and fractures, which can be found in a wide variety of ways. The literature begins to address this problem along with the first defects in the chassis axles. A review of recent research shows that researchers are looking to find the most optimal design solutions based on durability, lightness of construction, strength, and low cost. It also examines the causes of defects by evaluating the chemical composition of the metal, microstructure, mechanical properties, acting on loads, the location of the object under investigation in the whole system, and so on.

One of the most popular test methods used to detect cracks, fractures, and other defects is fractographic testing. Such investigations determine the complex causes of decay: the influence of the shape of the part, the load conditions, the interaction of the elements, the peculiarities of the structure of the material, and other factors are determined from the fractures.

In addition to traditional qualitative fractography, quantitative fractography aims to measure the topographic features of the fracture surface, revealing significant fracture surface characteristics. Modern fracture image analysis systems play an important role in advancing and successfully achieving these goals, not only to speed up measurement procedures but also to perform operations that would not be possible in other ways [18].

Fractography is a method in failure analysis for studying the fracture surface of materials. The nature of decay and fracture is characterized by specific characteristics of the mechanisms of decomposition (brittleness, plasticity, etc.). Each substance has the property of decomposing. As the material is exposed to various loads and environmental influences, the nature of decomposition is usually multifaceted. The nature of fracture of a part and the loading conditions has an interface that is characterized by: the nature of the fracture, the mechanism of crack formation, and the relief of the fracture surface [19].

Due to the high load on the rear axle, especially on the tractor, its service life is shortened. Fractographic studies show that the main cause of axle failure is fatigue. Fatigue cracks have been observed near welds. The results show that the axles break due to bending fatigue caused by improper welding [20,21]. Improper welding in the heat-affected zone (HAZ) reduces the plasticity of the material, resulting in structural stress concentration points and inclusions that subsequently affect the cracks. Pre-treatment of pre-weld and post-weld heat treatment of medium carbon steel is necessary to control the hardness level of HAZ and to reduce residual stress [20].

In the literature, it is often recommended to start the engineering calculations of axes from an analytical theoretical model, which can be used to estimate displacements, specific deformations, stresses, and the self-frequency spectrum [1,22]. Theoretical calculations of axle strength are one of the most important tasks in vehicle design. The axles are exposed to different external loads with a certain frequency, which depends on the speed, the actual load, the road conditions, and many other factors. At the same time, resonant phenomena are possible, which can lead to higher than nominal stresses and many other adverse phenomena. Variable external loads cause periodic changes in stresses, which contribute to the growth of fatigue cracks leading to fatigue fractures [1,23].

During the operation of the axle, the load acting on the axle housing in the vertical direction has a significant effect on the fatigue life of the components [24]. Cracks are caused by a constant stress concentration in the axle housing, resulting in fatigue at the concentration point. If the load exceeds a certain threshold, microscopic cracks will begin to form at the stress concentration point. Later, the cracks grow due to the cyclic load of fatigue. Eventually, the cracks reach a critical limit and then the structure suddenly breaks [21,25,26,27,28]. Axle housing failures are also affected by factors such as uneven load effect, housing slope, and eccentricity.

The paper presents an analysis of damage to the rear axle of a truck semi-trailer. For this purpose, FEA preceded by a theoretical introduction was used. Several load cases were considered in order to determine the most likely point of damage initiation to the semi-trailer axle. The structure of the work is as follows: the part covering materials and methods is presented in Section 2. Then, in Section 3, the results of analytical calculations and FEM numerical simulations for axle loads with various excitation (forces or displacement) are presented. Macroscopic analysis of the damaged element and material properties tests are also presented in Section 3. As a result of the tests and analyses carried out, conclusions were developed, which are included in Section 4.

## 2. Materials and Methods

### 2.1. Analytical Calculations

The first step in the analysis of the damaged semi-trailer axle was analytical calculations, which were then used as input data for dynamic stress analysis, which was then performed using the finite element method. The calculated values of individual parameters were obtained using appropriate mathematical relationships, taking into account the assumptions of engineering knowledge and the experience contained in the available professional literature was used. The results of the analysis are presented in Section 3.1.

### 2.2. FEM Simulations

**Figure 1:**
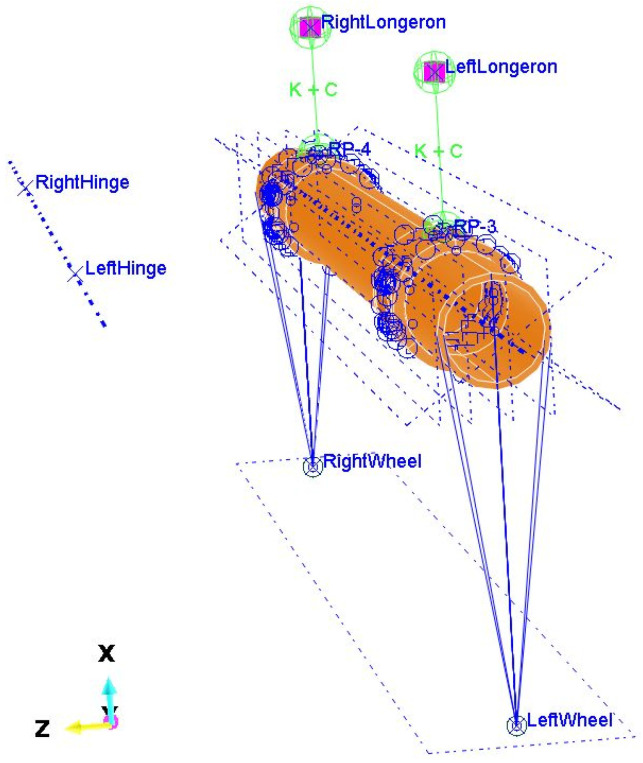
Complete model of the axle for simulations (front in Z direction).

Simulations of the operation of the semi-trailer’s non-driven axle were carried out using the Abaqus Explicit module. The model (Figure 1), apart from the axle itself, included two hinges, two bushings, two spring-damping elements and two points reflecting the contact points of the wheels with the road. The distribution of forces adopted for the simulation assumed an even position of the nominal load over the entire space of the platform.

The prepared model contains simplifications compared to the actual structure [29,30], i.e., the possibility of deformation of the wishbones, which were modeled with Rigid Body elements, was omitted. All degrees of freedom were fixed but with rotation around the Y axis. Each of the wishbones is coupled with a bushing that is mounted on the axle. This coupling also does not take into account the possibility of deformation of these two parts (rocker arm and bush) relative to each other. The bushings are connected to the side members by means of integrated spring/dashpot elements, while in reality the shock absorber works between the swingarm and the front side member, and the air bellows between the swing arm and the side member behind the axle. The model does not take into account the stiffness and damping of the road wheels. The contact points of the wheels with the road have been connected with Coupling elements to the ends of the axles.

In the presented model, the connection of the bushing with the axle tube was modeled using welds with a shape and location similar to the real structure. In addition, contact was modeled between the inner surfaces of the bushing and the outer cylindrical surface of the axle with a friction coefficient of 1.0. It was assumed that the spring elements of the suspension have a stiffness of 4.5 kN/mm and the damping coefficient of each shock absorber is 10 N s/mm. These parameters were selected in such a way that the wheel does not lose its grip with the ground and that the deflection of the suspension after its load corresponding to the permissible axle load is in the range of 15–20 mm.

**Figure 2:**
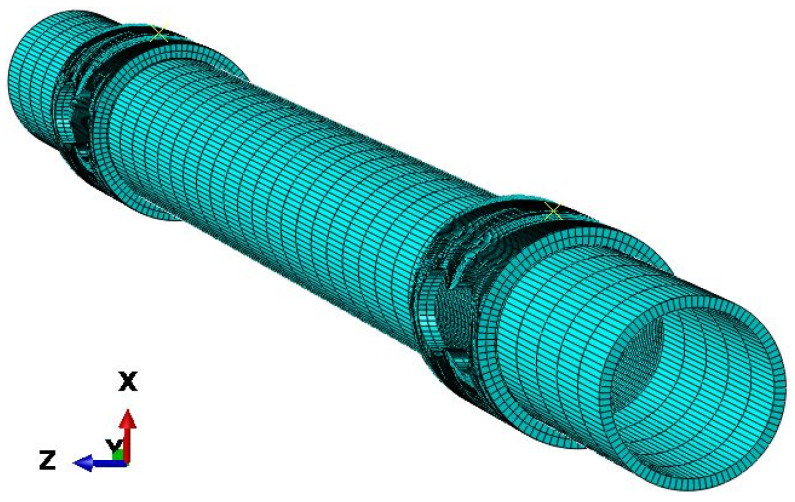
Axle meshed for simulations (front in Z direction).

The mesh for the axles and bushing was created using linear elements of the C3D8R type, which are bricks with an integration point reduced to its center. In the area of contact between these two parts, the mesh of elements was densified (Figure 2). The bushing has 11,496 elements and 17,943 nodes, while the axle has 10,120 elements and 20,424 nodes.

Simulations were carried out for eight axle load cases. For the load with the force of the wheels (wheels) in each case, the value of 44.1 kN was used, resulting from the nominal load capacity of the axle, which increases in 8 ms. A vehicle traveling at a speed of 50 km/h covers about 110 mm of road in this time.

One of the ways of loading concerned only the left wheel, i.e., as if the right wheel fell into a hole in the road. In the second case, both wheels were loaded simultaneously. Four consecutive cases consisted of vertical loading of both wheels, followed by: braking of the left wheel, braking of both wheels simultaneously, side loading of the left wheel, side loading of both wheels. The adopted values of forces (44.1 kN for each wheel) reflect the situation as if the coefficient of friction between the tires and the road surface was 1.0 both when braking and when cornering. One-wheel braking and lateral force on only one wheel can occur when the other wheel loses grip with the road surface.

Simulations were also carried out of the case when the left wheel overruns a small triangular mogul with a height of 30 mm, and then both wheels simultaneously overcome such an obstacle. Each of these cases was simulated as a kinematic excitation occurring in 8 ms.

The simulation results were analyzed in terms of the distribution of stresses and deformations of the axle material and compared with analytical calculations. Next, the results of the simulations (HMH max stress value, point/ node of max stress generation, number of stress cycles in the simulation time for max stressed nodes) were validated with the initiation point of the fracture observed on the real object.

### 2.3. Fracture Analysis and Fractography

The damaged semi-trailer axle was inspected. For the fractographic analysis, a fragment of the material from the damaged place was taken, then this element was cleaned of dirt and rust using the ultrasonic method. Fractographic analysis of the crack surface was performed using the MBS-2 stereoscopic microscope. Photographic documentation of the breakthrough was prepared using the Optikam Microscopy (Ponteranica, Italy) digital USB camera. The test sample was cut using a liquid-cooled Optimum (Long Island City, NY, USA) band saw. The sample was then subjected to further analyses.

### 2.4. Strength Tests

The obtained material samples from the damaged semi-trailer axle were subjected to a static tensile test. The tests of the mechanical properties of the steel were carried out in the Laboratory of Strength Mechanics of the Vilnius University of Technology in accordance with the procedure contained in the ISO 6892-1 standard. Young modulus was calculated as a regression coefficient. Tests for obtaining the curve were conducted according to ISO 6892-1:2019 annex G. The Instron 8801 servohydraulic fatigue testing system (with dynamic extensometer) was used for the mechanical characterization of the sample material.

## 3. Results and Discussion

### 3.1. Static Analyses of the Strength of the Axle Material

Bending around the horizontal axis was described in [31] and the results concerning bending around horizontal axis stresses are used here.

Additional bending due to potholes around the horizontal axis is investigated.

**Figure 3:**
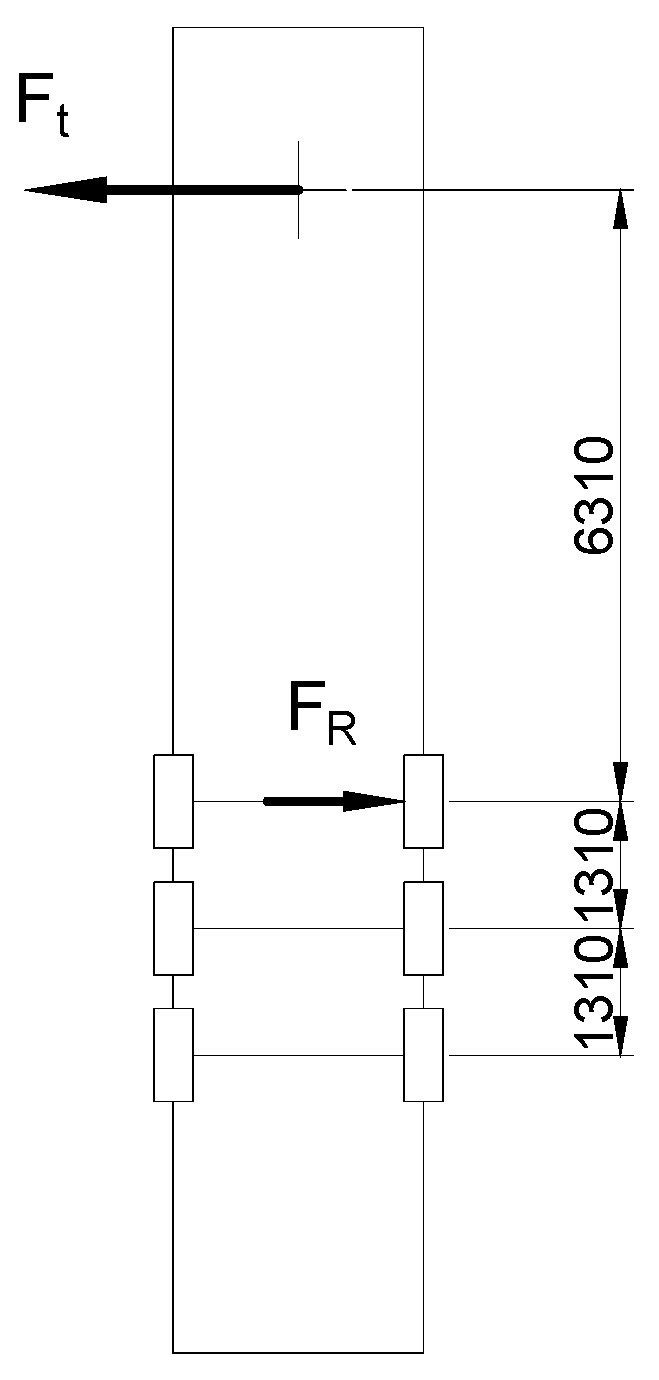
The scheme of the trailer turning.

If the truck goes to the left (or to the right), the wheel could be in way of the pothole. The most dangerous case in Figure 3 was found to be when the trajectory of the truck has less curvature. Therefore, the speed of the track is minimal. In this case there could be an additional bending moment acting around the horizontal axis, which could cause the bending moment to increase or decrease.

Pull force is the force that a truck or prime mover can exert onto a transporter, or any type of trailer for that matter.

To go from pull force, many terms and conversion factors are thrown on the table, including the number of driven axles, gearbox ratio, rear end ratio, tire size, truck weight, and fifth wheel capacity.

The track pool force according to [32] is 11.5 Mg for truck axles and for truck trailer axles it is no more than 9.0 Mg.

(1) $$F_{R}=9.0\cdot 1000\cdot 9.81\cdot \frac{1.0}{2}=44.1\;\mathrm{kN},$$

**Figure 4:**
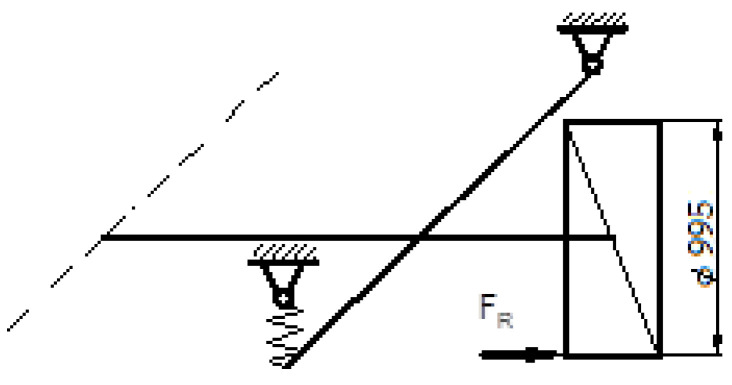
Reaction force.

The reaction force of the wheel (Figure 3) is derivable from the equation of equilibrium. The additional bending moment acting around the horizontal axis is shown in (Figure 4).

(2) $$M_{H}=44.1\cdot \frac{0.995}{2}=22.0\;\mathrm{kNm},$$

Load bending for one side of the axle:

(3) $$M_{L}=9.0\cdot 1000\cdot 9.81\cdot 262.5=23.2\;\mathrm{kNm},$$

(4) $$\sigma_{L}=\frac{M_{L}}{W}=\frac{23.2\cdot 10^{3}}{125\cdot 10^{-6}}=185.0\;\mathrm{MPa},$$

The full stresses due to bending moment acting around the horizontal axis:

(5) $$\sigma_{A}=\frac{M_{H}+M_{L}}{W}=\frac{\left(22.0+23.1\right)\cdot 10^{3}}{125\cdot 10^{-6}}=360.7\cdot 10^{6}\;\mathrm{Pa}=360.7\;\mathrm{MPa},$$

**Bending around the vertical axis**

The bending moment acting around the vertical axle is the result of the sudden braking of the truck. The important fact is that the most significant movement occurred just after the first intensive braking [31].

The inertia force of the truck for longitudinally forward, when braking, according to standard EN 12195-1 [32], is *F_i_ = m*·*g*·0.8.

For a semi-trailer, it is approximately 55% load [31] and one axle inertia load is equal:

(6) $$F_{1\mathrm{side}}=\frac{m\cdot g}{2}=\frac{9000\cdot 9.81}{2}=44.1\cdot 10^{3}\;N=44.1\;\mathrm{kN},$$

Especially, this internal force is decreasing in dynamic shock–image a wheel in a pothole during the braking of the truck. The authors accepted the dynamics coefficient according to [33] as being approximately equal to 1.2.

Bending moment acting around a vertical axis during braking:

(7) $$M_{V}=44.1\cdot 0.515\cdot 1.2=27.3\;\mathrm{kNm},$$

where: 0.515 m from the center of the wheel to the center of the rocker arm, (49.2 kNm taking into account the braking force).

Stresses due to bending moment around the vertical axis during braking:

(8) $$\sigma_{V}=\frac{M_{V}}{W}=\frac{27.3\cdot 10^{3}}{125\cdot 10^{-6}}=218.2\cdot 10^{6}\;\mathrm{Pa}=218.2\;\mathrm{MPa},$$

This value will be used for the calculation of compound stresses of the axle for finding the critical region. Torque from load and braking and stress

$$M_{LV}=\sqrt{M_{L}^{2}+M_{V}^{2}}=\sqrt{{\left(23.1\cdot 10^{3}\right)}^{2}+{\left(27.3\cdot 10^{3}\right)}^{2}}=35.8\;\mathrm{kNm}$$

(9) $$\sigma_{V}=\frac{M_{LV}}{W}=\frac{35.8\cdot 10^{3}}{125\cdot 10^{-6}}=286.1\cdot 10^{6}\;\mathrm{Pa}=286.1\;\mathrm{MPa}.$$

**Bending stresses**

The bending moments for tubular section may be summed up superimposed.

(10) $$\sigma_{B}=\frac{\sqrt{M_{H}+M_{V}}}{W}=\frac{\sqrt{{\left(22.0\cdot 10^{3}\right)}^{2}+{\left(27.3\cdot 10^{3}\right)}^{2}}}{125\cdot 10^{-6}}=280.1\cdot 10^{6}\;\mathrm{Pa}=280.1\;\mathrm{MPa},$$

The bending stresses are maximal in the front down quarter (Figure 3) of the axle.

**Torsion of the axle**

In a scenario with potholes, the wheels of the truck are on different levels. According to [34], air cushion throw is up to 410 mm, while in [35], the nominal ride height is, on average, 300 mm.

**Figure 5:**
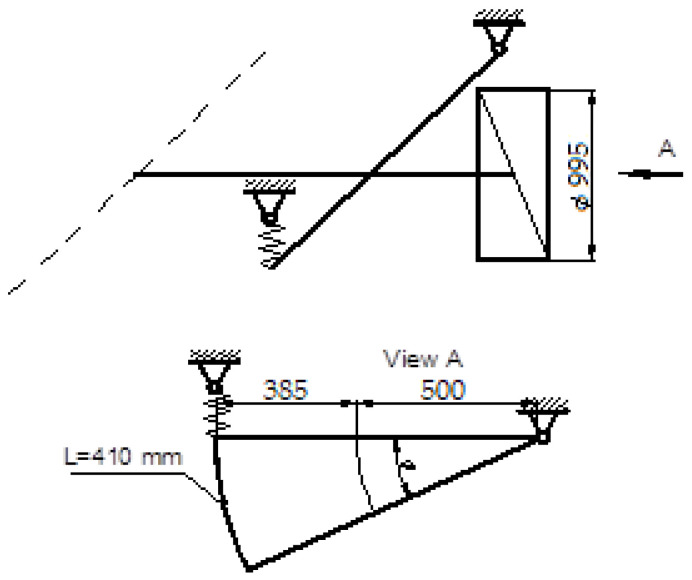
Air cushion throw, (L ≤ =300 mm [35]).

When one wheel is in the background and another is in the pothole, this results in torsion of the axle (Figure 5). The angle of twist is:

(11) $$\phi =\frac{200}{500+385}=0.339\;\mathrm{rad},$$

The angle of twist, internal torque, polar moment, length, and shear modulus are related by the formula:

(12) $$\Delta \phi =\frac{T\cdot l}{G\cdot I_{p}},$$

where: $I_{p}=2\cdot I=2\cdot 912=1824{\;\mathrm{cm}}^{4}$.

Shear modulus is obtained from a relationship that exists between *G*, *E* and *υ* (Poisson’s ratio).

(13) $$G=\frac{E}{2\cdot \left(1+\upsilon \right)}=\frac{204}{2\cdot \left(1+0.30\right)}=78.5\;\mathrm{GPa},$$

where *E*—modulus of elasticity (*E* = 204 GPa); *υ*—Poisson’s ratio (*υ* = 0.30).

Internal torque:

(14) $$T=F_{R}\cdot 0.500=44.1\cdot 0.5=22.1\cdot 10^{3}\mathrm{Nm}.$$

Shearing stresses in slow motion:

(15) τ=TWp=TIpde2=22.1⋅103250058=88.3⋅106 Pa=88.3MPa.

Shearing stresses in fast motion:

(16) τ=1.2⋅88.3=105.9 MPa,

Stress in axle when trailer crosses potholes:

(17) $$\sigma_{p}=\sqrt{\sigma_{L}^{2}+3\cdot \tau_{F}^{2}}=\sqrt{185.0^{2}+3\cdot 105.9^{2}}=260.6\;\mathrm{MPa},$$

**Compound stresses**

The influence of bending and torsion with a truck’s turning in slow motion is of an equation:

(18) $$\sigma_{d}=\sqrt{\sigma_{B}^{2}+3\cdot \tau^{2}}=\sqrt{280.1^{2}+3\cdot 105.9^{2}}=334.9\;\mathrm{MPa},$$

A compound dynamic stress of 335 MPa is calculated for driving at a speed 90 km/h on roads with potholes and more turns.

### 3.2. Effects of Simulations

As a result of the simulation, it was found that most often the area of maximum stress (HMH) in the axle material occurs in the place of its cooperation with the sleeve, where it is connected by a weld. This maximum lies closer to the wheel, on the outside of the axle, not between the wishbones. In addition, it usually occurs in the rear part of the axle, which, as it is further away from the axis of rotation of the wishbones, is exposed to higher torsional stresses when the deflection of the axle ends is different.

**Figure 6:**
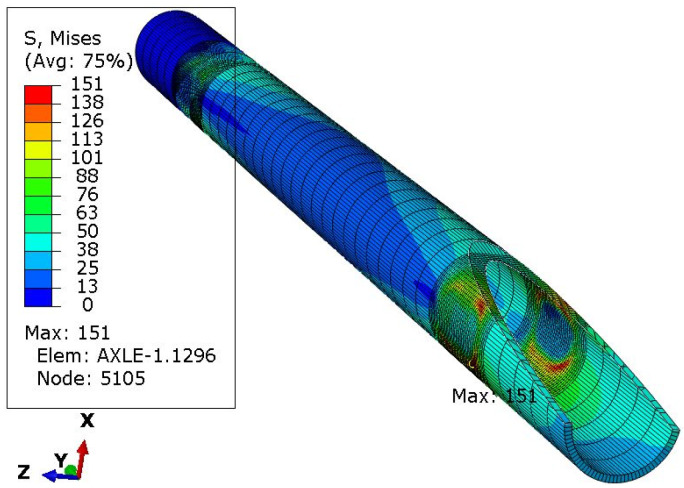
HMH stress distribution in the axle while only left wheel loaded with $F_{1\mathrm{side}}$ force. Trail front in Z direction.

**Figure 7:**
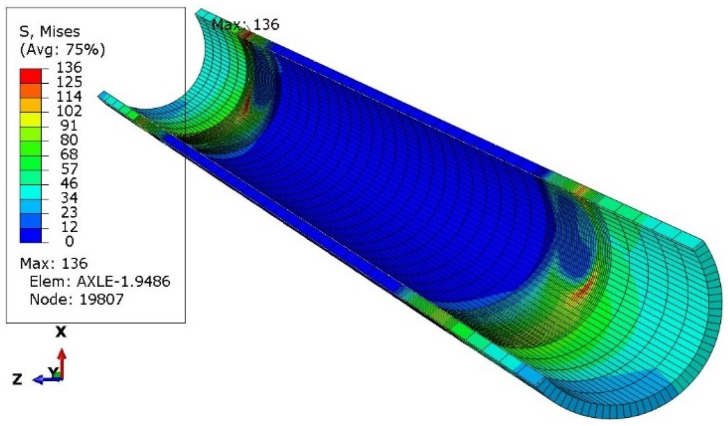
Stress distribution in the axle while both wheels loaded with $F_{1\mathrm{side}}$ force.

**Figure 8:**
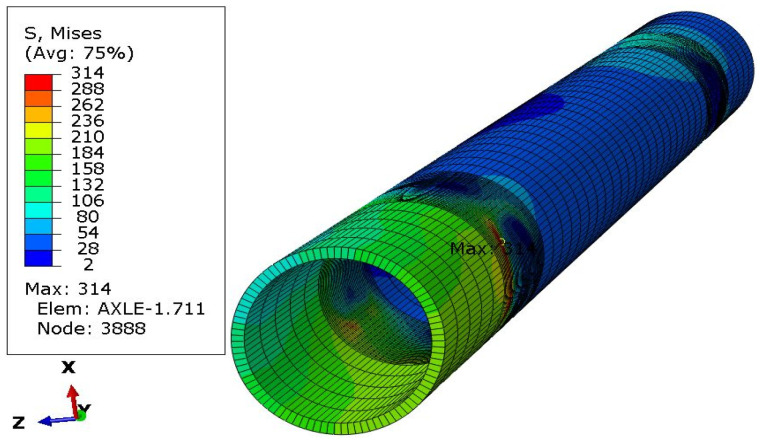
Stress distribution in the axle while only left wheel break is active.

**Figure 9:**
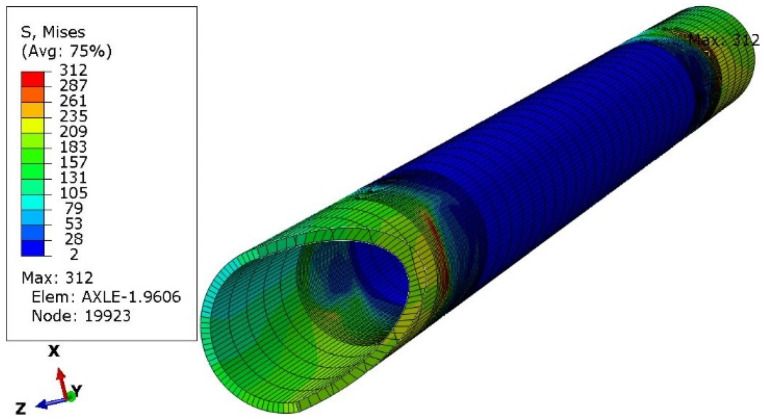
Stress distribution in the axle while only both wheels break.

**Figure 10:**
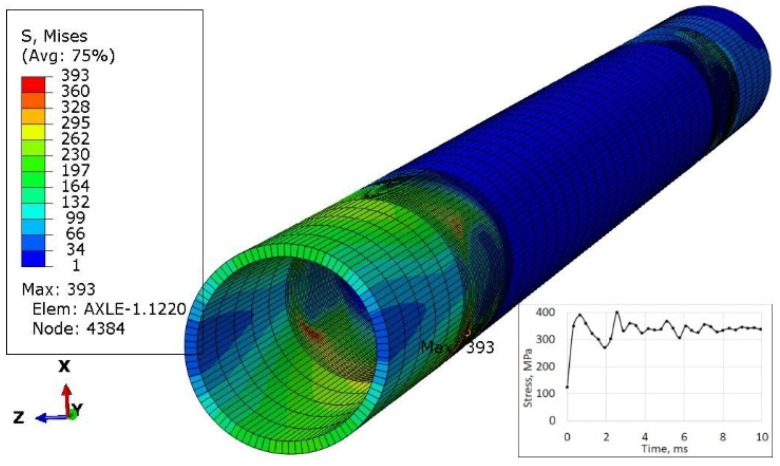
Stress distribution in the axle and max strengthen node 4384 while side force during turning acts to the left wheel.

**Figure 11:**
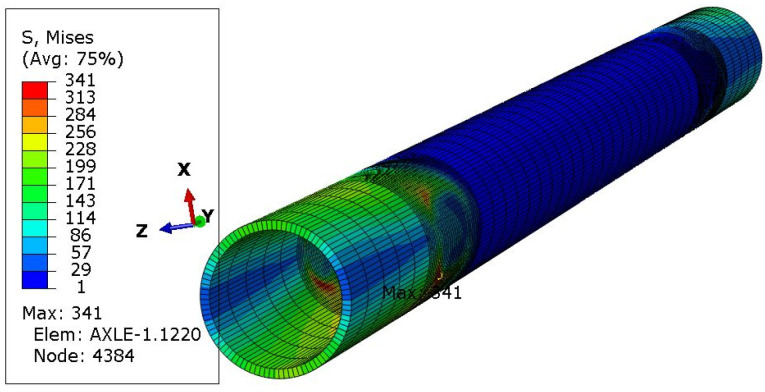
Stress distribution in the axle while side force during turning acts to both wheels.

One of the ways of loading (Figure 6) concerned only the left wheel, i.e., as if the right wheel fell into a hole in the road. Four subsequent cases concerned the vertical load on both wheels (Figure 7), followed by braking the left wheel (Figure 8), braking both wheels (Figure 9), side load on the left wheel (Figure 10), and side load on both wheels (Figure 11). The adopted values of forces ($F_{1\mathrm{side}}=44.1\;\mathrm{kN}$ for each wheel) reflect the situation as if the coefficient of friction between the tires and the road surface was 1.0, both when braking and when cornering. One-wheel braking and lateral force on only one wheel can occur when the other wheel loses grip with the road surface.

The analysis of the distribution of stress in Figure 6, Figure 7, Figure 8, Figure 9, Figure 10 and Figure 11 demonstrates that the max values in bushing were always near the welding. For the max strengthened node, the lowest value 136 MPa was observed for the symmetrical loading of two wheels (Figure 7). A slightly larger value (151 MPa) was found for the load on one (left) axle wheel. After that, when side (Figure 8 and Figure 9) or break (Figure 10 and Figure 11) force was added to the simulations, for the most loaded nodes, HMH stress was found to be at least twice as big (312 MPa).

**Figure 12:**
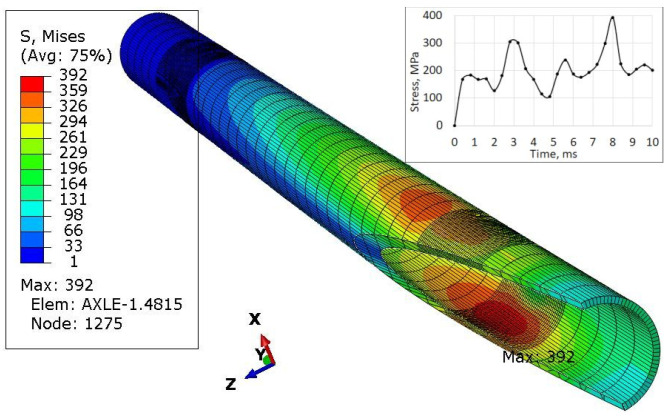
Stress distribution in the axle and max strengthened node 1275 while left wheel passes the triangular road deformation.

**Figure 13:**
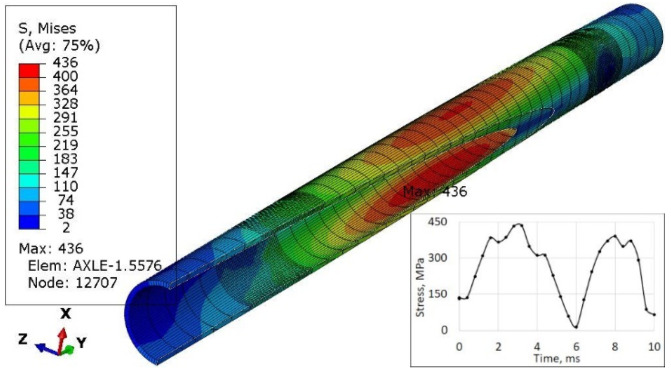
Stress distribution in the axle and max strengthened node 12,707 while both wheels pass the triangular road deformation.

The last simulations concerned the case where the left wheel overruns a small trough (Figure 12) of a triangle shape with a height of 30 mm, and then both wheels simultaneously overcome such an obstacle (Figure 13). Each of these cases was simulated as a kinematic excitation occurring in 8 ms.

When forcing the vertical displacement of the left wheel, the maximum stresses occur at the point of contact between the sleeve and the axle in the lower part of the axle (Figure 12). It can be seen from the graph that apart from the average stresses in the range of 100–200 MPa, there are sudden jumps in their values (peaks) by about 200 units to over 300 MPa and many load cycles.

The value of stresses in the axle (436 MPa in Figure 13) for the case of symmetrical excitation of both wheels is higher than for the displacement of only one wheel (392 MPa in Figure 12). However, their course over time is less abrupt for node 12,707 (Figure 13) than for node 1275 (Figure 12).

For the excitation carried out as displacements of both wheels, the highest stresses of 436 MPa were recorded. They occurred in the central part of the axis, with its deformation corresponding to the first form of natural vibrations.

For analytical calculations, each case can be considered separately: load, braking, side forces.

### 3.3. Visual Fractographic Analysis

**Figure 14:**
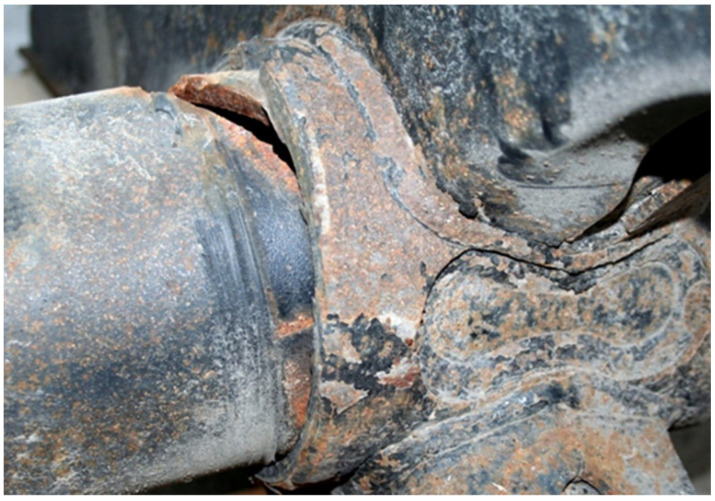
Axis fracture location.

The test object is a broken axle of a semi-trailer chassis. The axle broke in February 2016. The total mileage of the axle is 326,516 km. The axle breaks near the weld (near the mounting location). The broken axle is analyzed in the condition in which it was delivered for service under warranty. After inspecting the axle, a fracture of the axle is visible near the coupling joint in which the axle is fastened with the bracket. Non-destructive methods show only part of the fracture (Figure 14). Fractographic analysis of the fracture surface was performed using the MBS-2 stereo microscope and an Optikam Microscopy digital USB camera.

For fractographic analysis, a specimen is excised from the fracture site. The ultrasonic bath removes dirt and rust from the fracture surface before inspecting the specimen. The specimen was cut using an Optimum liquid-cooled band saw. Liquid cooling during cutting was used to prevent the structural transformation of the steel.

Mechanical properties of steel tests were performed in the Laboratory of Strength Mechanics of Vilnius Gediminas Technical University according to standard ISO 6892-1. For further analysis using FEM (Finite Element Method), a numerical model of the considered trailer axle was prepared and simulations were carried out using the Abaqus Explicit module.

**Figure 15:**
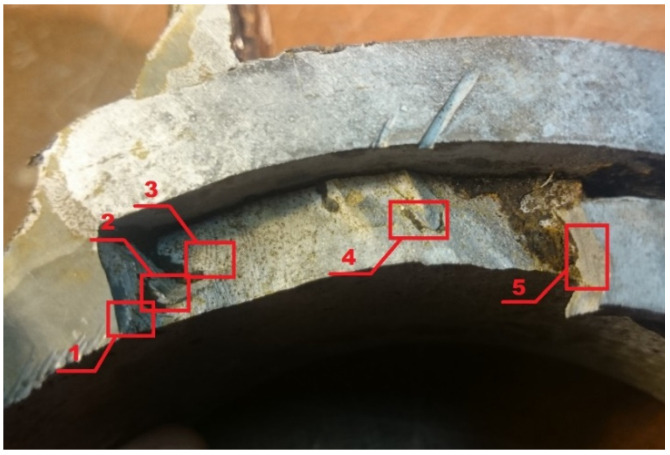
Fracture surface: Areas 1 to 5 are used for fractographic analysis.

Fractures of a complex nature predominate in the fracture as the structural material is subjected to environmental influences and various deformations (Figure 15). In the manufacturing process, for example in the welded joints, areas of different mechanical properties are formed. Where there is a decrease in the plasticity of the material or in the area of higher stresses, individual voids may appear and a process of long-term plastic deformation may begin. By inspecting the fracture surface, three areas of fracture can be distinguished: fatigue before fracture, the onset of fracture, and major fracture.

**Figure 16:**
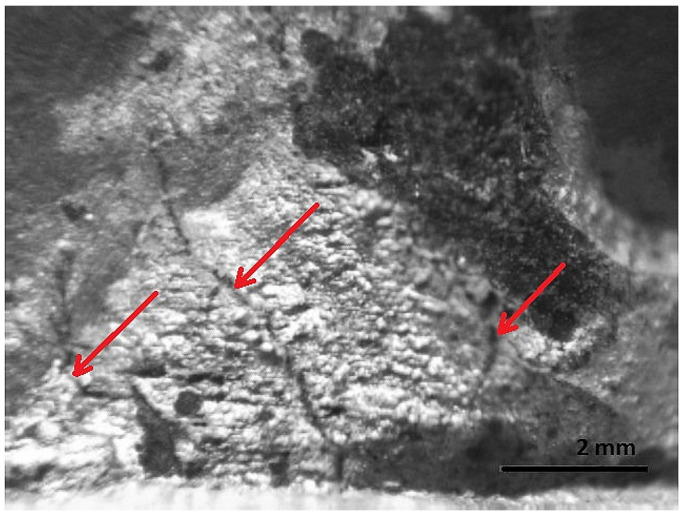
Fracture area: the area of the focal point of decay.

**Figure 17:**
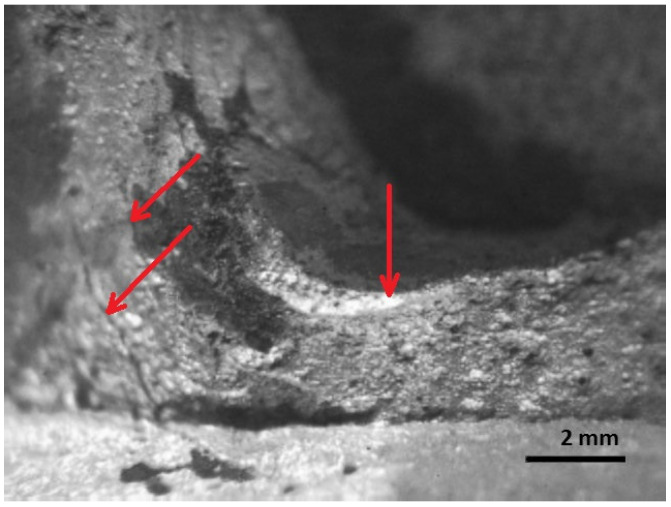
Fracture area: wider view of the area of the focal point of decay.

The first is the area of fatigue before fracture or decay; it has a wavy relief (Figure 16 and Figure 17).

In the area of fatigue before the fracture, in the base and at the weld metal, near the weld where the highest stresses are applied, cracks are observed and a fragile fine-grained structure is visible. The formation and propagation of a crack depending on the type of deformation, the structure of the material, the level of the load, the shape of the part, and many other factors. The fracture is thought to have been initiated by long-term loading, as cracks and ribbed fracture morphology are characteristic of the effects of long-term loading.

**Figure 18:**
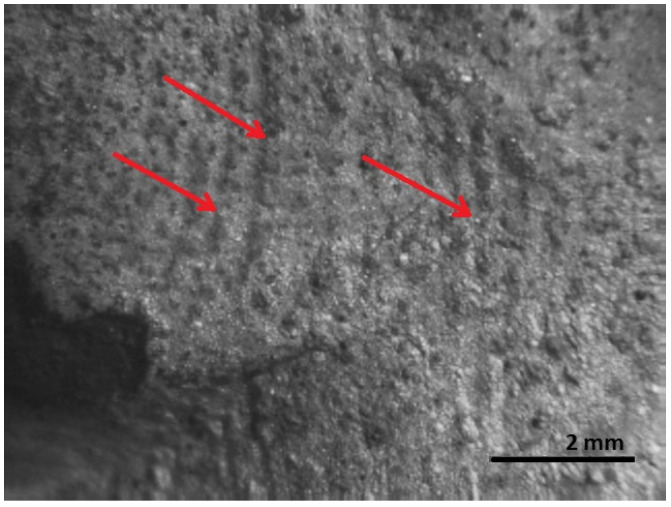
Fracture area: the area of gradual crack growth.

**Figure 19:**
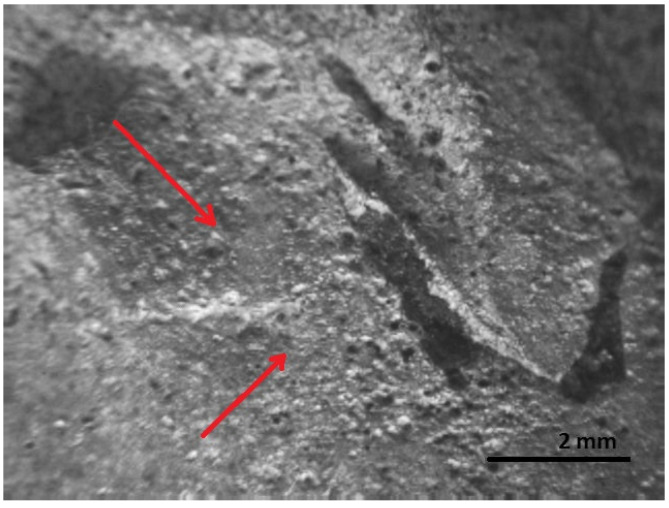
Fracture area: The area of the final fracture.

**Figure 20:**
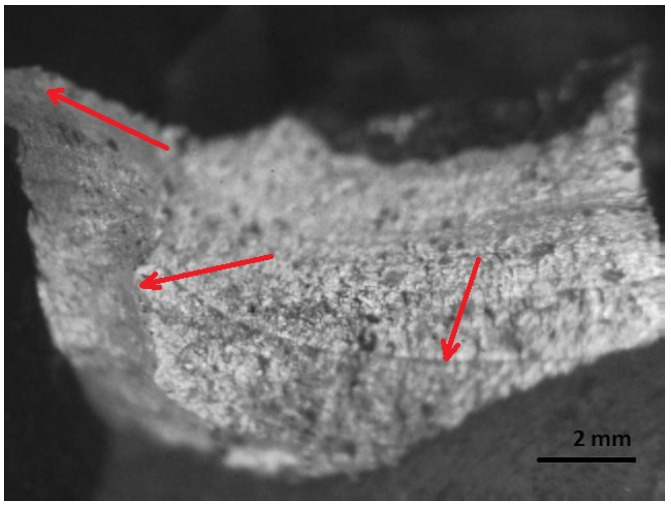
Fracture area: area of plastic deformation.

Figure 16, Figure 17, Figure 18, Figure 19 and Figure 20 show the results of fractographic studies of individual fracture areas (Figure 15). Fracture surfaces: Figure 16—an area no. 1; Figure 17—an area no. 2; Figure 18—an area no. 3; Figure 19—an area no. 4; Figure 20—an area no. 5.

The accumulation of plastic deformation occurs during long-term operation. It is known that during plastic deformation, the density of dislocations in the metal increases. New dislocations are due to new internal sources, the best known of which is the Franco-Reed source. The increase in the density of dislocations affects the constant increase in the hardness of the metal. To achieve this, the process of increasing the hardness of the metal is accompanied by an increase in the brittleness of the metal. As a result, micro-cracks appear on the surface in the area of maximum hardness. During subsequent operations, these cracks turn into macro-cracks.

Fatigue fracture occurs when the surface of the fracture is perpendicular to the direction of the maximum stresses and has characteristic areas: the first—the decay foci, the second—the gradual increase in the crack, the third—the final fracture. In the area of the decay foci, a fine crystalline relief structure is visible, in the second area, the relief is crystalline in structure, and the third area is also crystalline in structure but fragile. Cracks are noticeable on the surface of the part.

The second is the area at the beginning of the fracture. Grooves and fatigue thresholds are observed, which are characteristic of low-cycle fatigue fractures (Figure 18). Fatigue thresholds and grooves are among the signs of macroscopic decay. These signs indicate the direction of crack propagation, which is associated with plastic deformations, a decrease or increase in the rate of crack propagation, depending on the effects of the environment.

The relief of the main or final fracture area is crystalline in structure, brittle, and the surface is rough and porous (Figure 19).

If all materials were absolutely plastic or absolutely brittle, plastic, or brittle fracture would occur during the tensile tests. Since there are no absolutely plastic and absolutely brittle materials, structural plastic materials are fragile when they decompose.

At the edges of the fracture, the shear characteristic of plastic deformation is seen, as well as grooves and fatigue thresholds characteristic of fatigue fracture (Figure 20).

Fractographic analysis shows that it is a fatigue fracture characterized by three fracture areas and the fracture surface oriented perpendicular to the direction of maximum stresses. It can be assumed that the fracture may have been initiated by a long-term load.

### 3.4. Strength Properties of the Axle Material

**Figure 21:**
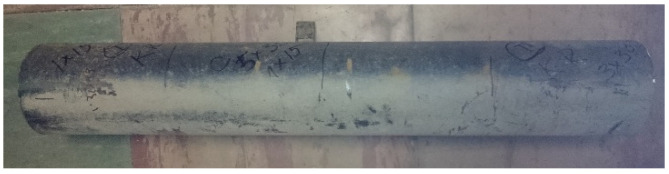
Tested middle part of axel.

The mechanical properties of the steel of the axle were obtained experimentally according to standard ISO 6892-1. The middle part of the axle was used for testing (Figure 21).

**Figure 22:**
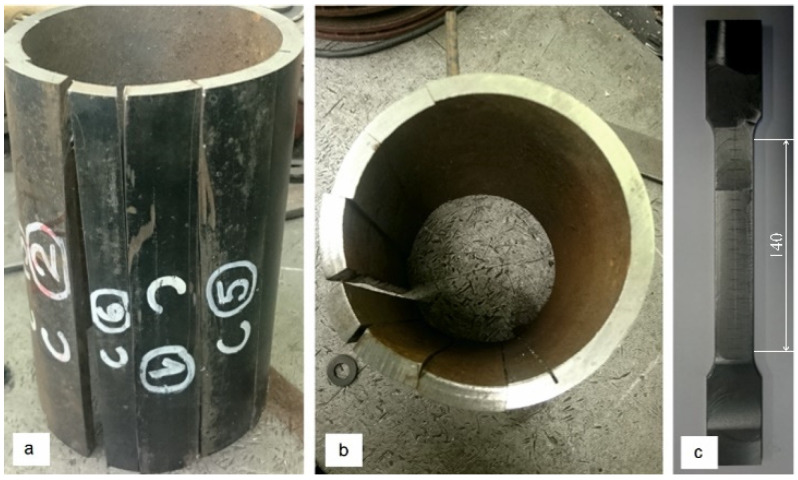
The blanks for specimens: (**a**) number of blanks; (**b**) orientations of blanks (**c**) specimen.

The blanks for specimens were cut from the middle. It was important to know the numbers of blanks and their position in the axle (Figure 22a,b). The specimen for the tension test was produced by the milling process (Figure 22c).

To identify the cross sections area of the specimens (Table 1), the cross sections parameters such as length *a* and thickness *b* were measured. The Young’s modulus *E =* 204 GPa, yield stress $\sigma_{y}=$ 581 MPa and ultimate stress $\;\sigma_{u}=$ 663 MPa (Table 1) were obtained as average from the experimental curves.

The axis in this place puts the difficult working conditions of the surface in contact with the sleeve. This can cause corrosion of the axle surface (compare Figure 14 and Figure 15) and fretting. In addition, the end of the axle is located near the wheel, from which sand and gravel can be thrown onto the surface of the material and damage the paint coating. Corrosion pits cause additional weakening of the material and surface notches, which are dangerous, especially when the material is subjected to fatigue, i.e., in the conditions of axle operation. The safety factor related to the min yield stress value obtained from strength tests is 567/392 = 1.45. It should be noted that the result of 567 MPa was obtained for a standardly prepared tensile sample (Figure 22c). On the surface of the axis, near the fracture (Figure 14), corrosion pits are clearly visible, reducing the strength of the material, and causing geometrical and structural stress concentrations.

Poisson’s ratio *ν* = 0.30, which is standard for steel was taken.

**Figure 23:**
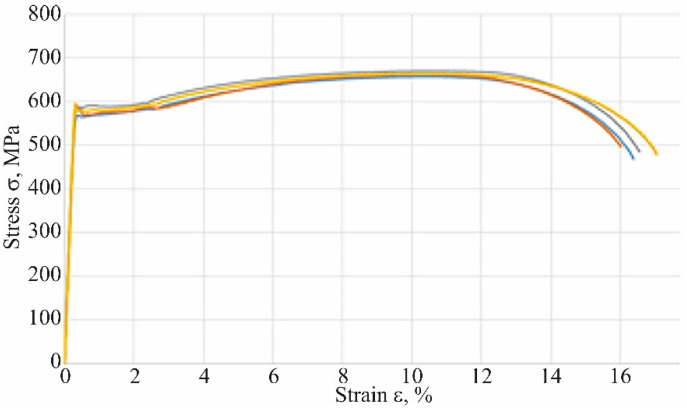
Experimental stress–strain diagrams.

After tensions, tests of the specimens were measured and machine diagrams force F- displacement L were transformed to stress σ–strain ε diagrams. Finally, the behavior of the steel used for the axle is described by the non-linear stress σ–strain ε diagram (Figure 23), which is an average of results.

In any case, this result has shown that about 60% (335/567) of elastic behavior of steel is used. Note that the analysis Equation (18) did not evaluate possible stress concentration factors due to welding impact on an axle and damage to its surface by corrosion (compare Figure 14).

In addition, the surface of the central part of the pipe is well protected with an anti-corrosion coating (Figure 21) and there is no danger of its abrasion in contact with the sleeve (compare Figure 1) or the impact of gravel thrown by a wheel rolling nearby. For these reasons, a safety factor of 567/436 = 1.30 (Figure 13) may be sufficient to achieve permanent fatigue strength.

Table 2 shows that only in the case of the simplest analytical calculations (bending stress from permissible load for one side of the axle $\sigma_{L}$) were the calculated stresses greater than those resulting from the simulation. In all other cases, it was the alternative: the simulation resulted in stresses higher than those calculated analytically. The reason for this may be that the simulation takes into account all types of axle loads, while the analytical calculations (except $\sigma_{p}$) did not take into account possible torsion (different deflection of the wishbones).

Apart from the case of overcoming a hole in the road, the absolute difference between the stress values calculated and those resulting from the simulation was about 30 MPa, i.e., about 5% of the yield stress value.

## 4. Conclusions

On the basis of the non-destructive tests as well as analyses and simulations, the following conclusions were made.

1. Fractographic analysis shows that it is a fatigue fracture characterized by three fracture areas and the fracture surface oriented perpendicular to the direction of maximum stresses.
2. It can be assumed that the fracture may have been initiated by a long-term load, as cracks and ribbed fracture morphology are characteristic of the effects of long-term loading.
3. The mechanical characteristics of the steel of the axle are obtained experimentally: the Young’s modulus *E* = 204 GPa, lowest yield stress $\sigma_{y}=$ 567 MPa and average ultimate tensile stress $\sigma_{u}=$ 663 MPa.
4. In the critical region of the axle during slow turning of the track, bending stresses reach 507 MPa in a dangerous quarter of the cross section. It consists 87% of yielding of steel. Respectively, in fast drive, it consists of 49%.
5. When driving the truck fatly on roads with potholes, due to the resulting torque, the compound stresses reach 335 MPa. However, 40% of the reserve is left up to the yield stress of the steel, but corrosion pits in the contact area of the sleeve and bushing may decrease the value.
6. Analytical solutions have shown that even when the truck is turning on bumpy roads, the yield strength is exceeded and the 93% strength limit is reached. This inevitably raises the fracture in the critical load impact region of the axle.
7. Stress obtained during simulations for the max loaded nodes was usually bigger than calculated and more realistic. It may be caused by the fact that the analytical calculations did not take into account possible torsion as an effect of different deflection of the wishbones while driving.

## Figures and Tables

**Table 1:** Mechanical characteristics of axle.

Specimen	*E*, GPa	$\sigma_{y}$, MPa	$\sigma_{u}$, MPa
C01	196	567	657
C02	203	592	662
C03	208	572	670
C04	207	592	664
Average	204	581	663

**Table 2:** Comparison of maximum stresses calculated analytically (A) and at simulations (S).

Result of	Stress, MPa			
	$\sigma_{L}$	$\sigma_{A}$	$\sigma_{LV}$	$\sigma_{p}$
A	185	361	286	261
S*	151	393	314	391
S	136	341	312	436
A-max(S *,S)	34	−32	−28	−173

* load (wheel displacement) of left side only.

## Data Availability

Not applicable.
